# Host–Pathogen Interactions in Measles Virus Replication and Anti-Viral Immunity

**DOI:** 10.3390/v8110308

**Published:** 2016-11-16

**Authors:** Yanliang Jiang, Yali Qin, Mingzhou Chen

**Affiliations:** State Key Laboratory of Virology and Modern Virology Research Center, College of Life Sciences, Wuhan University, Wuhan 430072, China; 2013202040037@whu.edu.cn

**Keywords:** measles virus, paramyxoviruses, viral replication, host factors

## Abstract

The measles virus (MeV) is a contagious pathogenic RNA virus of the family *Paramyxoviridae*, genus *Morbillivirus*, that can cause serious symptoms and even fetal complications. Here, we summarize current molecular advances in MeV research, and emphasize the connection between host cells and MeV replication. Although measles has reemerged recently, the potential for its eradication is promising with significant progress in our understanding of the molecular mechanisms of its replication and host-pathogen interactions.

## 1. Introduction

Measles, also known as rubeola or morbilli, is a contagious infection caused by measles virus (MeV), an RNA virus of the genus *Morbillivirus* within the family *Paramyxoviridae* [1]. Species of this genus also include the canine distemper virus in canines, rinderpest virus in cattle, and morbilliviruses which infect other animals [2]. Humans are the natural hosts of MeV, and no animal reservoirs are known to exist [3].

The virion of MeV is a pleomorphic or spherical particle with a diameter ranging from 120 to 1000 nm and has two major structural components: one is the helical ribonucleoprotein (RNP) core formed by the association of the nucleoprotein (N), phosphoprotein (P) and large protein (L) with the viral genome, the other is the cellular membrane-derived lipid envelope surrounding the RNP core [1,4,5]. The active RNP complex is responsible for initiating primary transcription after cell entry as well as counteracting the host interferon (IFN) signaling pathway [5,6,7]. The MeV RNP is bound by the matrix protein (M), and then covered by the lipid envelope containing two spike glycoproteins, F and H proteins, which are primarily responsible for membrane fusion and receptor attachment, respectively [8].

For measles, symptoms usually develop after an incubation period of 7–14 days and last 7–10 days. They typically include fever, cough, coryza, conjunctivitis, enanthema (Koplik spots) on the oral mucosa, and a maculopapular rash. Apart from the typical symptoms, a specific feature of measles is a long-lasting immunosuppression due to the loss of immune memory B and T cells [9]. As a consequence, patients can encounter complications, especially in the setting of malnutrition in developing countries, ranging from bacterial superinfections, pneumonia, and diarrhea to postinfectious encephalomyelitis (PIE), or a sub-acute sclerosing panencephalitis (SSPE), which can manifest even several years after recovery [10,11,12]. Vaccination is highly effective and has resulted in a huge decrease in measles-related mortality [4,12,13]. Measles, however, has not been eliminated and has even reemerged in developed countries owing to a low vaccine coverage rate; therefore, more efforts are needed for a worldwide elimination of MeV [14,15].

The aim of this review is to outline recent progress in the MeV research that is critical to understanding MeV replication and the virus–host biology. Here, we emphasize the findings related to the MeV genome, function of viral proteins, replication cycle, and the involvement of host cell factors that play key roles in the MeV replication cycle.

## 2. Genome and Lifecycle

### 2.1. Genome

Currently, 24 MeV genotypes compiled in eight clades (A–H) have been recognized by sequencing 450 nucleotides (nt) that code for the *C*-terminal 150 amino acids of the *N* gene [16,17]. However, cross-neutralization with strain-specific antisera revealed only one serotype [18]. Following the rule of six, the RNA genome of MeV is 15,894 bp in length and is tightly encapsidated by the helically arranged N protein to form a helical nucleocapsid (NC) containing N-RNA that is also observed in other *Paramyxoviridae* members [19,20]. The genome begins with a 52 nt non-coding region known as the leader and ends with a 37 nt non-coding region known as the trailer, both of which are essential for the transcription and replication of the genome [21]. The organization of the MeV genome is similar to that of most other members of the *Paramyxoviridae*: there are six genes coding for eight viral proteins arranged as 3′-N,P,V,C,M,F,H,L-5′, each flanked by gene-end and gene-start sequences [1,22].

The first gene codes for N protein. The conserved *N*-terminal N core (about 400 amino acids) constitutes the core region of N protein whereas the remaining *C*-terminal N tail (about 100 amino acids) is intrinsically disordered and interacts with the matrix protein and the *C*-terminal domain of phosphoprotein [19,23,24]. Moreover, the structural flexibility of the disordered N tail is important for interactions between the N tail and multiple cellular proteins, including the 70 KDa heat shock protein (Hsp72), eukaryotic translation initiation factor 3 (eIF3-p40) and interferon regulatory factor 3 (IRF-3) [25,26,27].

The second gene codes for three proteins—P, V, and C—via an RNA editing process and an alternative reading frame [28]. The P protein binds to newly synthesized N to form a soluble N^0^–P complex, thus preventing N from binding to cellular RNAs, and the N^0^–P complex is used as a substrate for the specific encapsidation of viral RNA [29,30]. In addition, the P protein tethers the polymerase onto and progresses along the N-RNA template by binding to the NC [31,32,33,34].

The main function of the V and C proteins is to suppress the host innate immune response by interfering with IFN signaling pathways [1]. These proteins also function as virulence factors in that they are indispensable for virus infection in vivo [35]. For the related Sendai virus, the C protein even enhances the release of the M protein in a manner dependent on the endosomal sorting complexes required for transport (ESCRT) pathway [36].

The third gene codes for the M protein, which is a hydrophobic protein. Although M is not a membrane protein, it associates with membranes, probably through its hydrophobic surface [37]. It also binds RNPs, associates with the cytoplasmic tails of F and H proteins and modulates cell fusion [24,38]. In addition, it acts as an inhibitor of viral polymerase activity, affecting both mRNA transcription and genome replication [24,39]. Thus, the M protein plays a crucial role in many stages of the viral lifecycle.

The fourth and fifth genes code for envelope-associated spike glycoproteins indicated in membrane fusion and receptor recognition, that are discussed below in the “Assembly and Egress” subsection later in this article. The last gene codes for the RNA-dependent RNA polymerase (RdRP), which is believed to possess all catalytic functions required for RNA synthesis, including ribonucleotide polymerization, capping and methylation, and polyadenylation [40,41]. The L, N, and P proteins associate with the viral RNA to form the active RNP complex that initiates primary transcription after cell entry [42,43].

### 2.2. Cell Entry

The initial binding of MeV to the cell surface is mediated by the tetrameric H protein via interaction with cell surface receptors, which triggers the conformational change of the trimeric F protein and then, the membrane fusion and the delivery of the viral RNP core into the cytoplasm [44,45]. Similar to the H proteins of the Morbillivirus, the H protein of MeV cannot bind sialic acid and lacks neuraminidase activity; thus, it is named H not HN [1,46,47]. The primary receptors for wild type MeV strains are CD150/SLAM and nectin-4/PVRL4 [48,49], and some laboratory-adapted and vaccine strains bind to CD46 as well. In addition, the F protein is critical for the fusion of infected cells with neighboring cells, which eventually results in multinucleated cell formation, termed “syncytia”, which is a hallmark of MeV and many other paramyxoviruses [8,50,51,52].

### 2.3. Transcription and Replication

MeV shares the gene order and transcription strategy that are fundamental characteristics of all other paramyxoviruses [22,53]. Following cell entry, the genomic RNPs are released into the cytosol and the encapsidated viral RNA serves as a template of the RdRP complex for both transcription and replication [5]. Transcription begins at the 3′ end of the genome and viral genes are transcribed in the 3′ to 5′direction with a sequential “stop–start” mechanism. MeV shares the gene order and transcription strategy that are fundamental characteristics of all other paramyxoviruses [22,53]. Newly synthesized viral mRNAs are translated to viral proteins by using the host translation machinery. The negative-strand genome is also used to synthesize a positive-strand anti-genome, which is a complementary copy of the entire genome that produces more genomes via the same viral RNA polymerase. During replication, the newly synthesized genomic RNA is tightly wrapped with the N protein to provide a helical template for viral transcription and replication [32,54]. Although the mechanism of the switch from transcription to replication remains unclear, evidence suggests that the accumulation of N proteins is critical for it [42].

### 2.4. Assembly and Egress

The assembly of the M protein, the RNP complex, and the glycoproteins at selected sites on the plasma membranes of infected cells lead to the formation of fully infectious MeV particles, which is a result of coordinated interactions between viral components as well as between viral and cellular factors [45,55]. The *C*-terminal domain of the N protein has been proved to be essential for the interaction with the M protein by yeast two-hybrid binding assay and co-immunoprecipitation in mammalian cells [24], and mutations or deletions in the *M* gene block the transport of RNP complex to the plasma membrane during infection, further supporting the crucial role of M protein in incorporating the RNP complex into virions [56,57]. In addition, the M protein can assemble to form higher structure, and binds cellular membranes and cellular factors as well [45,55,58,59]. Consequently, it is generally considered the key driver of paramyxovirus particle assembly and budding. For many paramyxoviruses, including MeV, M protein expressed in the absence of other viral proteins is sufficient to form virus-like particles [37,56]. While many viruses take advantage of the cellular ESCRT machinery during egress, the budding of MeV particles is driven primarily by the M protein, which has been demonstrated to be ESCRT-independent [60,61].

Taken together, the aforementioned findings suggest that, for MeV, the F and H proteins assemble intracellularly prior to receptor binding and are co-transported to the plasma membrane. The M protein associates with the RNP complex in the cytoplasm and then carries it to the plasma membrane, where the assembly with F and H proteins occurs. Fully infectious virions are then released from the host cell in an ESCRT-independent manner.

## 3. Interaction between MeV and Cellular Factors

### 3.1. Host Factors Involved in MeV Replication

#### 3.1.1. Host Factors Involved in MeV Entry

As an obligate intracellular parasite, MeV interacts with numerous cellular molecules to manipulate cellular processes and to subvert anti-viral responses for its replication. At the entry level, at least three cellular receptors have been identified for wild type strains and laboratory-adapted strains. The primary receptor for wild type strains is CD150/SLAM, the expression of which is limited to activated T and B cells, macrophages and dendritic cells [48,62]. CD150/SLAM supports the transport of the infection to lymphocytes and results in temporary loss of immunity to other pathogens, which accounts for MeV-induced immune suppression [63]. The epithelial cell receptor nectin-4/PVRL4 was identified later using a comparative microarray approach [49,64]. In addition to the two aforementioned receptors, the laboratory-adapted and vaccine strains use CD46, which is expressed on nearly all nucleated cells [65,66]. Consequently, using CD46 as an additional receptor results in a tropism alteration of MeV. Despite the lymphocyte and epithelia cell tropism of wild type MeV strains, MeV may also infect other types of cells. For example, PIE and SSPE are complications of MeV infection that result from the infection of neurons [67,68]. Thus far, the mechanism accounting for MeV neuronal infection and transport is unclear.

#### 3.1.2. Host Factors Involved in MeV RNA Synthesis and Assembly

Many putative host factors involved in MeV RNA synthesis have been identified via yeast two-hybrid, co-immunoprecipitation and some proteomic approaches. Some findings have suggested that the inducible Hsp72 can directly modulate RNA synthesis of several mammalian RNA and DNA viruses, including MeV [69,70]. During RNA synthesis, P protein binds to two conserved hydrophobic domains on the *C*-terminal N tail to tether the RdRP to the ribonucleocapsid. By competing with P protein for binding to these domains, Hsp72 would loosen the binding between RdRP and the ribonucleocapsid so that RdRP can move to the next N tail, which sustains the RdRP processivity, resulting in increased genome transcription, replication, and virulence [25,71]. In addition, another host factor, SHC binding and spindle associated 1 (SHCBP1), was found to interact with both the C and the P proteins, and they did not compete for the binding [72]. The results indicated that the C protein interacted with the RNP complex through SHCBP1 and thereby modulated viral RNA synthesis. Without the SHCBP1-binding site, however, the C protein retained the regulatory ability, suggesting that other cellular factors might have similar functions as SHCBP1. Besides just exploiting the regulation on itself, MeV inhibits the translation of cellular mRNA via the interaction between the p40 subunit of eIF3 and the N protein [26].

Other host factors that interact with MeV proteins include several kinases, such as casein kinase II that phosphorylates P protein and some unidentified kinases that phosphorylate N and P proteins [73,74]. Phosphorylation of these two proteins has totally different functions. Phosphorylation of P protein at S86 and S151 downregulates viral transcriptional activity, whereas MeV RNA synthesis is increased upon the phosphorylation of serine residues 479 and 510 in N protein, as confirmed [75] in a mini-genome expression system [76,77]. 

**Figure 1:**
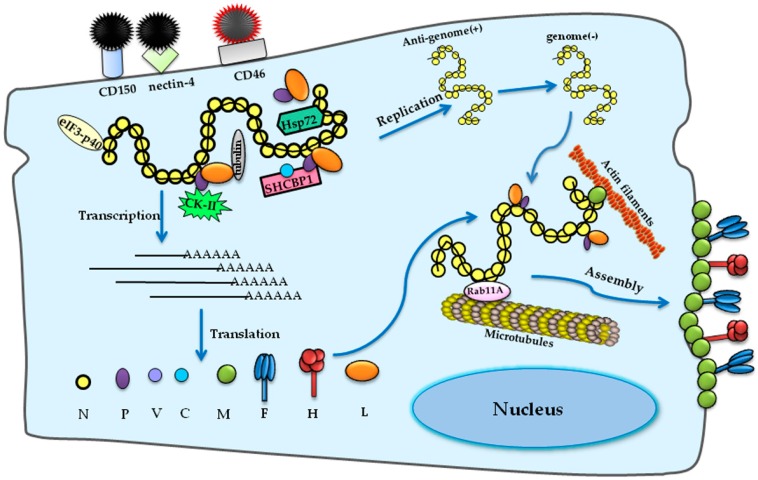
Schematic of host factors involved in the measles virus (MeV) lifecycle. The attachment and entry of MeV is mediated by H and F proteins, associated with cellular receptors CD150, nectin-4 for wild-type strains and CD46 for attenuated strains, respectively. During viral RNA synthesis, heat shock protein 72 (Hsp72) interacts with the N protein to sustain the RdRP processivity. The C protein interacts with the RNP complex through SHC binding and spindle associated 1 (SHCBP1) and modulates viral RNA synthesis. The N protein can also bind to eukaryotic translation initiation factor 3 (eIF3-p40) to inhibit the translation of cellular mRNA. There are some kinases that phosphorylate N and P proteins, including the casein kinase II (CKII). As for assembly and budding, the RNP complex is transported to the plasma membrane driven by the M protein, and the process is dependent on actin filaments and microtubules associated with Ras-related protein Rab11A.

Many viruses take advantage of the cellular trafficking system during their replication. Cytoskeletal tubulin has been shown to be an essential component for the transcription and replication of Sendai virus and vesicular stomatitis virus [78,79]. In MeV, tubulin also acts as a positive factor that it may be a subunit of the RdRP complex, resulting in the subsequent RNA synthesis [80]. Moreover, the MeV RNP complex is transported in a microtubule-dependent manner associated with recycling endosomes containing Ras-related protein Rab-11A [81]. Together with microtubules, another cytoskeleton component, actin, is essential for the reproduction of MeV as well, especially for budding. Experimental data revealed that the accumulation of RNP and defects in the maturation and release of infectious MeV particles were connected with the disruption of actin filaments and that actin filaments were packaged within the virions, suggesting a close association between actin filaments and MeV assembly and budding [75,79,82]. These host factors interacting with MeV during its lifecycle are summarized in Figure 1.

### 3.2. Host Factors Involved in Anti-MeV Innate Immune Responses

#### 3.2.1. Host Factors Involved in IFN Response

The host has innate immunity, in particular the IFN system, to sense and protect it from MeV infection. However, MeV has also evolved multifaceted strategies to antagonize the immune attack.

The innate immune responses are activated by the sensing of pathogen-associated molecular patterns (PAMPs) via pattern recognition receptors (PRRs), including retinoic-acid inducible gene (RIG)-I-like receptors (RLRs), Toll-like receptors (TLRs), and nucleotide-binding oligomerization domain (NOD)-like receptors (NLRs), that play an essential role in MeV detection [83,84]. The host cell can recognize the H, N proteins, and RNA of MeV [7,83,85]. For example, the interaction of H protein with Toll-like receptor 2 (TLR2) triggers the production of interleukin 6 (IL-6) and the expression of SLAM/CD150 on the cell surface, resulting in immune activation and effective spreading of MeV [85]. Following cell entry, the viral RNA is released into the cytoplasm and recognized by two key members of the RLR family: RNA helicase-like RIG-I and melanoma differentiation-associated protein 5 (MDA5) [83,84,86].

MeV has evolved multifaceted strategies to counteract viral RNA sensing. Like V proteins of other related paramyxoviruses, the V protein of MeV interacts with MDA5 and RIG-I-like receptor 2 (LGP2) at the same time, and the latter has been reported as both a coactivator of MDA5 and a negative regulator of both RIG-I and MDA5 [87,88,89]. By interacting with phosphoprotein phosphatase 1 α and γ (PP1α/γ), V protein can also prevent PP1-mediated activation of MDA5 [90]. Moreover, P protein suppresses the TLR4 signaling via the activated transcription of a ubiquitin-modifying enzyme A20, which negatively regulates the nuclear factor-kappa B (NF-κB) [91,92].

Sensing of MeV infection activates signaling cascades resulting in the activation of the transcription factors NF-κB and IRF-3/7, leading to the production of type I IFN-α/β and proinflammatory cytokines [84,93,94]. To antagonize the induction of IFN, MeV has evolved multiple mechanisms. The V protein binds to IκB kinase α (IKKα) to downregulate the phosphorylation of IRF-7, and it also inhibits the transcriptional activities of IRF3 and IRF7 by interacting with them [95,96]. Moreover, V protein interacts with and suppresses NLR family member NLRP3 inflammasome-mediated IL-1β secretion [97]. The P, V, and C proteins can bind and inhibit NF-κB-dependent gene expression, especially the binding of the V protein with NF-κB subunits p65 [98]. Despite the P, V, and C proteins-induced suppression of NF-κB signaling, MeV-induced activation of NF-κB pathway, which was crucial to its replication, was reported to be regulated by sphingosine kinase 1 (SK1) [99]. The data revealed that overexpression of SK1 promoted the replication of MeV. However, the inhibition of SK1 suppressed both the MeV replication and the MeV-induced NF-κB signaling, suggesting the dual role of NF-κB and the pro-viral role of SK1 in MeV replication. The mechanism of how the NF-κB signaling promotes MV replication needs to be further explored, such as the exact stage of the MeV lifecycle where SK1 initiates the regulation. The C protein impairs the activation of the protein kinase regulated by RNA (PKR) via regulating the production of defective copyback double-stranded viral RNA [100]. In addition, C protein of wild-type MeV can also counteract the promoter of IFN-β in the nucleus without inhibiting the activation of IRF-3. However, the C protein of vaccine strains cannot localize into the nucleus, suggesting that altered C protein intracellular localization of vaccine strains contributes to its attenuation [101]. On the contrary, the N protein interacts and activates IRF-3, which stimulates IFN-β production [7]. However, a direct interaction between IRF-3 and N could not be confirmed experimentally by using purified recombinant proteins and yeast two hybrid assay: this finding led to the hypothesis of an indirect binding requiring a specific cellular context [27].

**Figure 2:**
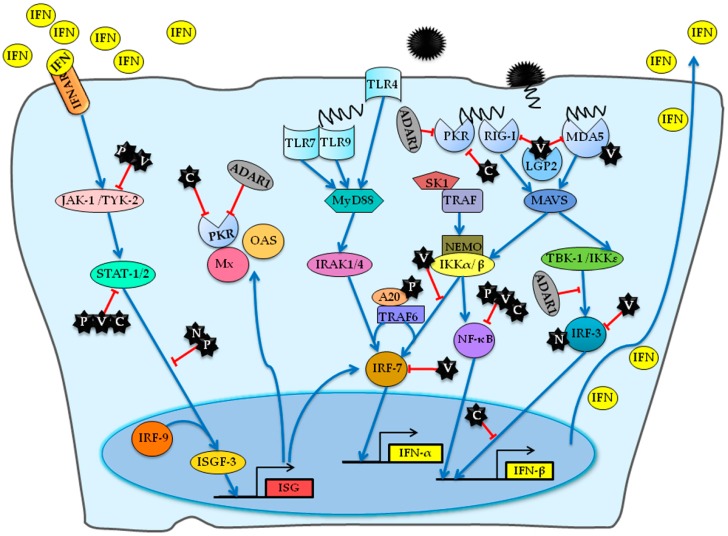
A detailed overview of host factors involved in MeV-stimulated interferon (IFN) induction and signaling. The C and V proteins inhibit the activation of the viral RNA sensors retinoic-acid inducible gene I (RIG-I), melanoma differentiation-associated protein 5 (MDA5) and RIG-I-like receptor 2 (LGP2) and the following IFN responses. Furthermore, V protein inhibits the activation and function of interferon regulatory transcription factor 3 (IRF-3) and IRF-7 via interaction with them. The P, V, and C proteins bind subunits of nuclear factor-kappa B (NF-κB) and repress the production of IFN-β and cytokine. In addition, the interaction between P protein and the negative regulator A20 also leads to the repression of NF-κB. In addition, sphingosine kinase 1 (SK1) interacts with TNF receptor-associated factor 2 (TRAF2) and enhances tumor necrosis factor (TNF)-induced activation of NF-κB signaling. In the nucleus, C protein represses IFN-β transcription without inhibiting the activation of IRF-3. On the contrary, N protein positively activates the IRF-3 and IFN-β production. The interferon inducible protein ADAR1 negatively regulates the activation of protein kinase regulated by RNA (PKR) and the IRF-3, suppressing MeV-mediated IFN-β production. To interfere with the IFN signaling, C protein blocks the dimerization of phosphorylated signal transducer and activator of transcription 1 (STAT-1). P and V protein inhibit the phosphorylation and nuclear translocation of STAT-1. In addition, N protein can prevent the migration of STAT-1 and STAT-2 into nucleus. The IFN signaling triggers the expression of many IFN-inducible proteins. The arrows indicate the activation of IFN responses while the T-ended arrows indicate the repression by MeV. JAK-1, Janus kinase 1; TYK-2, tyrosine kinase 2; ISG IFN-stimulated gene; IRF, interferon regulatory factor; IKK, IκB kinase; TLR, Toll-like receptor; ADAR1, adenosine deaminase acting on RNA 1; MAVS, mitochondrial antiviral-signaling protein; OAS, 2′-5′-oligoadenylate synthetase; IRAK, interleukin-1 receptor-associated kinase 1.

MeV is capable of interfering with both IFN synthesis and the signal transduction pathway mediated by the release of IFN, thus allowing the successful escape of MeV from the innate immune system. Secreted IFN-α/β activates Janus kinase/signal transducers and activators of transcription (JAK/STAT) signaling in surrounding noninfected cells via binding to the type-I IFN receptors (IFNAR), thus triggering the expression of numerous IFN-stimulated genes (ISGs), some of which encode anti-viral proteins [102,103]. To inhibit the establishment of an antiviral state in non-infected cells, MeV has evolved a variety of evasive adaptions to counteract the JAK/STAT signalling pathway. For example, N and V proteins of MeV cause the defective nuclear redistribution of activated STAT [104,105]. The N-terminal and *C*-terminal regions of V protein interact with JAK-1, STAT-1 and STAT-2, which independently impair the signal transduction [106]. P protein inhibits the phosphorylation and nuclear translocation of STAT-1 [107,108], while C protein prevents the dimerization of phosphorylated STAT-1 [109]. Of note, the IFN-stimulated gene ADAR1 inhibits the activation of PKR and IRF-3, suggesting its function as a suppressor of MeV-mediated IFN-β production, which ultimately promotes MeV infection [110,111]. Furthermore, the activation of PKR can also be suppressed by the C protein of MeV [112,113]. The interactions involved in MeV-stimulated IFN induction and signaling are summarized in Figure 2.

#### 3.2.2. Host Factors Involved in Stress Granule Formation

In addition to the synthesis of IFN and the subsequent signaling cascades, another response of host cells to infection of several paramyxoviruses, including MeV, is the formation of stress granules (SGs), which are cytoplasmic aggregates containing various translationally stalled mRNAs, 40S ribosomes, and RNA-binding proteins [112,114,115]. In general, the activation of IFN system, especially the activation of PKR, results in the phosphorylation and inactivation eIF2α, leading to the formation of SGs [115]. By binding with the eIF3-p40, the N protein inhibits the translation of cellular mRNA, which may consequently promote SG formation [26]. Experiments with cells stably deficient either in PKR or adenosine deaminase acting on RNA 1 (ADAR1) showed that the formation of SG was induced in a PKR-dependent manner by MeV infection; however, SG formation was suppressed by ADAR1 [112].

Taken together, the aforementioned findings suggest that ADAR1 acts as a suppressor of host innate immune responses, including IFN-β production and SG formation induced by MeV infection. Of particular note, SG cannot form in cells infected by wild-type MeV; however, a mutant lacking C protein expression is a robust inducer of SG formation, which is consistent with the suppression of the activation of PKR by the C protein [112]. Given that SG formation cannot be induced by wild-type MeV infection, MeV may benefit by interfering with SG formation, suggesting an antiviral effect of SGs during MeV infection.

#### 3.2.3. Host Factors Involved in Autophagy

Autophagy is a highly conserved mechanism that mediates the dysfunctional cytoplasmic components for lysosomal degradation and recycling, whose hallmark is the formation of the double-membraned autophagosomes that sequently fuse with lysosomes to form autolysosomes for degradation. Autophagy not only maintains cellular and tissue homeostasis but also regulates innate immune responses against intracellular virus invasion [116]. Consequently, viruses have developed various mechanisms to escape or even hijack the autophagy machinery for their own benefit. For MeV, the infection can induce successive proviral autophagy signaling via different pathways, which ultimately promotes the formation of infectious viral particles [117].

Both virulent and attenuated strains induce a late and sustained autophagy wave, which is initiated after viral replication and relies on the expression of C protein of MeV. In addition, the expression of C protein alone can also induce an autophagic signaling in an immunity-related GTPase M (IRGM)-dependent manner in Hela cells [118]. The late autophagy signaling can be sustained overtime as a result of the formation of syncytia [117]. Moreover, the extensive syncytia formation mediated by the expression of both H and F proteins in cells expressing one of the cellular receptors is sufficient to induce autophagy. Therefore, the expression of C protein is not necessary to induce autophagy in syncytia [119].

As for attenuated strains being able to bind CD46, the infection can induce two waves of autophagy, including an early but transient autophagy wave via the engagement of CD46-Cyt-1 in a Golgi-associated PDZ and coiled-coil motif-containing protein (GOPC)-dependent pathway and the late autophagy wave [120]. Research findings have suggested that the involvement of CD46 in the autophagic digestion of MeV peptides and the subsequent presentation by MHC-II may explain the acquisition of protective immunity via vaccine strains of MeV infection [121].

Of note, MeV induces de novo formation of autophagosomes, and such autophagosomes mature into autolysosomes, whereas some paramyxoviruses make use of autophagy machinery to replicate but inhibit autophagosome maturation by blocking the fusion of autophagosomes with lysosomes [120,122].

**Figure 3:**
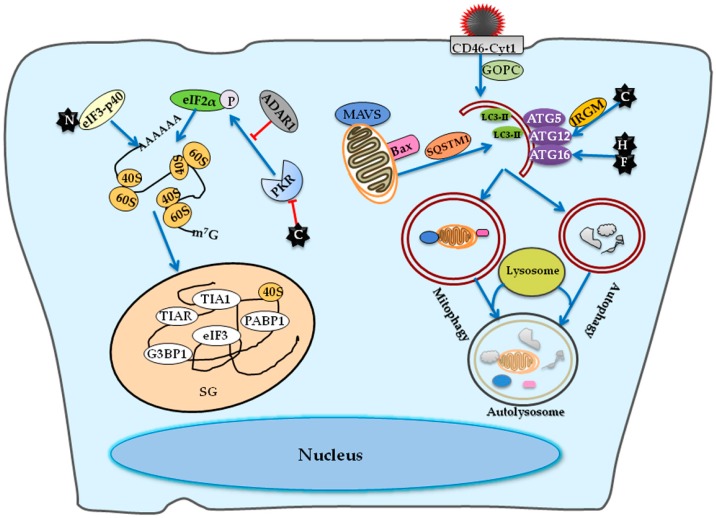
A diagram of host factors involved in MeV inducible stress granule formation and autophagy. The C protein suppresses the activation of PKR, so a mutant MeV lacking C protein expression rather than the wild-type MeV induces the stress granule (SG) formation. The N protein binds to the eIF3-p40 and then promotes SG formation. As a suppresser of PKR, ADAR1 suppresses the PKR-dependent SG formation. The infection of Edmonston-MeV strain initiates an early but transient autophagy wave via the engagement of CD46-Cyt-1 in a Golgi-associated PDZ and coiled-coil motif-containing protein (GOPC)-dependent pathway. Moreover, the expression of C protein can induce an autophagic signaling in an immunity-related GTPase M (IRGM)-dependent manner. The expression of H and F proteins in cells expressing one of the cellular receptors is also sufficient to induce autophagy. The Edmonston-MeV strain also exploits the selective mitophagy via recognition of damaged mitochondria by autophagic receptor SQSTM1, resulting in the degradation of mitochondrion-tethered mitochondrial antiviral-signaling protein (MAVS) and subsequent attenuation of RIG-I/MDA5-mediated responses. The arrows indicate the activation of innate immune responses while the T-ended arrows indicate the repression by MeV.

Apart from bulk autophagy, the Edmonston-MeV strain also exploits the selective mitophagy in non-small cell lung cancer (NSCLC) cells, which enhances its replication as well [123]. The mitophagy occurs upon Edmonston-MeV infection via recognition of damaged mitochondria by the autophagic receptor SQSTM1/p62, the devouring of these mitochondria by autophagosomes, and the subsequent degradation by lysosomes [123]. By clearing damaged mitochondria before they release cytochrome c, mitophagy prevents the beclin 1-mediated activation of Bid or the degradation of active caspase-8 and then inhibits apoptosis, resulting in migitated cell death and the consequent oncolysis in NSCLC induced by Edmonston-MeV [124]. More important than the inhibition of apoptosis, though, is the attenuation of the RIG-I/MDA5-mediated signaling pathway via the SQSTM1/p62-mediated degradation of mitochondrion-tethered mitochondrial antiviral signaling protein (MAVS), suggesting a novel mechanism of Edmonston-MeV to weaken the innate immune responses [123]. An overview of host factors involved in MeV inducible stress granule formation and autophagy is depicted in Figure 3.

## 4. Conclusions

In this review, we provide a summary of current achievements in MeV research and review the experimental efforts aimed at elucidating the molecular mechanisms of MeV replication and host–pathogen interactions. We focus on recent insights related to the MeV genome, the function of viral proteins, the replication cycle, and the involvement of host cell factors during the MeV lifecycle, with an emphasis on cellular factors mediating MeV-stimulated innate immune responses that play key roles in the MeV replication cycle.

The elucidation of the mechanisms of MeV infection has provided valuable information on viral replication and countermeasures to mitigate cellular innate immune responses, which will ultimately provide new targets for antiviral therapy against MeV. However, many details of the mechanisms remain unclear; therefore, further investigation is needed to provide a clearer and more comprehensive understanding of the aforementioned aspects.
